# Finite-Size Effects in Periodic EOM-CCSD for Ionization Energies and Electron Affinities: Convergence Rate and Extrapolation to the Thermodynamic Limit

**DOI:** 10.1021/acs.jctc.4c01451

**Published:** 2025-02-04

**Authors:** Evgeny Moerman, Alejandro Gallo, Andreas Irmler, Tobias Schäfer, Felix Hummel, Andreas Grüneis, Matthias Scheffler

**Affiliations:** † The NOMAD Laboratory at the FHI of the Max-Planck-Gesellschaft, Faradayweg 4−6, 14195 Berlin, Germany; ‡ Institute for Theoretical Physics, TU Wien,Wiedner Hauptstraße 8−10/136, 1040 Vienna, Austria

## Abstract

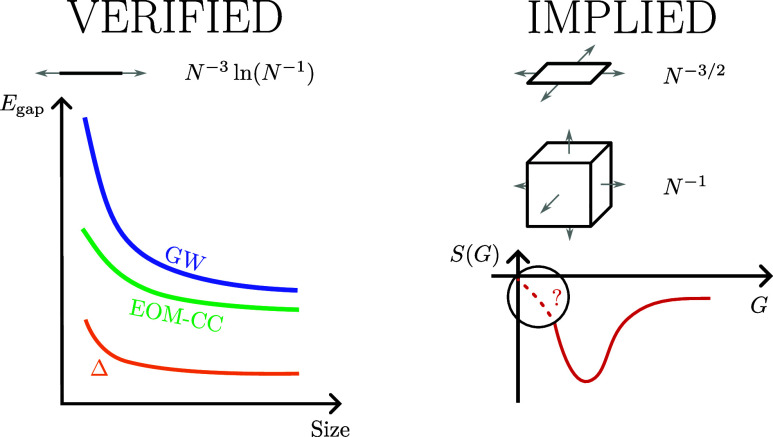

We investigate the convergence of quasiparticle energies for periodic systems to the thermodynamic limit using increasingly large simulation cells corresponding to increasingly dense integration meshes in reciprocal space. The quasiparticle energies are computed at the level of equation-of-motion coupled-cluster theory for ionization (IP-EOM-CC) and electron attachment processes (EA-EOM-CC). By introducing an electronic correlation structure factor, the expected asymptotic convergence rates for systems with different dimensionality are formally derived. We rigorously test these derivations through numerical simulations for *trans*-polyacetylene using IP/EA-EOM-CCSD and the *G*_0_*W*_0_@HF approximation, which confirm the predicted convergence behavior. Our findings provide a solid foundation for efficient schemes to correct finite-size errors in IP/EA-EOM-CCSD calculations.

## 1 Introduction

For most theoretical materials science studies, density functional theory (DFT) is employed due to its favorable balance between computational scaling and moderate accuracy. However, for many material properties the accurate inclusion of electronic exchange and correlation effects is critical to achieve a qualitatively correct description for scientifically and technologically important properties. In particular, for the theoretical description of electronic band gaps and band structures, most Kohn–Sham density functional approximations (KS-DFAs) in use today are known to generally severely underestimate band gaps, oftentimes referred to as the *band gap problem*.^1^ Relaxing the condition of a multiplicative exchange–correlation potential of KS-DFT to allow for a more flexible integral operator as it is the case in generalized KS-DFT resolves many of the issues associated with the band gap problem,^1^ generally reducing the band gap error and rectifying the missing derivative discontinuity. Apart from hybrid functionals, which are the most widely used representatives of generalized KS-DFAs, the electronic structure method of choice for the calculation of quasiparticle energies has become the *GW*-approximation,^2,3^ which takes the DFA one-electron wave functions as a starting point but explicitly accounts for electronic exchange and correlation effects using a perturbation theory approach. The *GW*-approximation yields significantly improved band gaps compared to the most widely used approximate density functionals. However, the most commonly used method based on the *GW* approximation, the *G*_0_*W*_0_ method, is known to have its limitations as well, the most glaring one being the dependency of the band gap result on the underlying DFA, the so-called *starting point dependence*.^3^ Other higher-order corrections to the *GW* approximation require the inclusion of so-called vertex corrections in the self-energy and the screened interaction *W*.^4,5^ Although certain improvements can be achieved using vertex corrections, it remains challenging to systematically improve upon the *GW* approximation, which already achieves an excellent trade-off between computational cost and accuracy.^6−8^

A systematically improvable method is the equation-of-motion coupled-cluster^9^ (EOM-CC) framework. EOM-CC theory, being the extension of ground-state CC theory to excited states, allows to theoretically describe systems upon removal (IP-EOM-CC), addition (EA-EOM-CC) or vertical excitation (EE-EOM-CC) of an electron. As—in the present convention—the electronic band gap is defined as the sum of the electron affinity/attachment (EA) and the ionization potential (IP), it is possible to obtain band gaps and entire band structures in the EOM-CC framework.^10,11^ The relation between EOM-CC and *GW* band gaps was also investigated, showing the differences and similarities between the diagrammatic contributions and the results of both approaches.^12−14^ However, the major obstacle of ab initio calculations employing EOM-CC theory, and CC theory in general, is the high computational cost and excessive memory requirements. In addition to that, CC theory explicitly incorporates long-range electronic exchange and correlation contributions, causing a slow convergence to the bulk-limit as will be discussed in the present work. Due to the high computational cost of CC methods, it is even more challenging than for DFA and *GW* calculations to converge to the thermodynamic limit (TDL), which is approached by increasing the number of particles *N*_part_ in the simulation cell, *N*_part_ → ∞, while keeping the particle density constant. The *GW* approximation partly ameliorates this problem by adding corrections for the long-range behavior of the dielectric function using *k*·*p*-perturbation theory, which are often referred to as head- and wing-corrections.^15^ In recent years, studies of electronic band gaps via quantum Monte Carlo (QMC) methods have been published as well,^16,17^ where finite-size effects were also discussed as one of the major sources of error. For these QMC band gaps, the *N*-electron ground-state and the electronic state with one electron more (*N* + 1) or less (*N* – 1) were determined separately and the energy difference of these states was computed to obtain the band gap value. As the leading-order contribution to the finite-size error, the interaction of the added particle (*N* + 1) or hole (*N* – 1) with its periodic images was identified, which was corrected by subtracting the screened Madelung term from the quasiparticle band gap. Higher-order finite-size errors resulting from multipole moments of the charged states were corrected by means of system size extrapolation. Unfortunately, these approaches are not straight-forwardly applicable to EOM-CC methods: The dielectric function, and therewith the head- and wing-correction to it, is not directly accessible in the canonical EOM-CCSD formulation. For that, linear response CC theory would be necessary.^18^ In contrast to the QMC ansatz, CC methods do not use trial wave functions but generally rely on the Hartree–Fock (HF) wave function as a starting point, which already incorporates the Madelung term. Instead, one needs to resort to extrapolation techniques or more sophisticated finite-size error estimations using the transition structure factor,^19,20^ which, however, has been only formulated for ground-state CC theory so far and is only a viable technique if the correlation structure factor is represented in a plane wave (PW) basis. Using a very different approach for the simulation of crystalline systems in the CC framework, it has been demonstrated that via a cluster embedding approach accurate band gap predictions can be achieved.^21^ It must be stressed, however, that the results discussed in the present work assume periodic boundary conditions. A second point of departure is that the work on band gaps from cluster embedding techniques utilized similarity transformed EOM (STEOM) theory, while we in this work explore the EOM-CC method. References (10, 11, and 22) report periodic IP/EA-EOM-CC calculations for solids using a Gaussian basis set. The computed band gaps have been extrapolated to the TDL assuming an *N*_*k*_^–1/3^ convergence rate. Here, we present a careful investigation of the reliability of the assumed convergence rate and the importance of next-to-leading order contributions to the finite-size errors.

In this work, all CC and EOM-CC calculations were performed using a super-cell approach. Even though it is entirely sufficient and—due to the exploitation of the translation symmetry—computationally significantly more efficient to calculate the band gap of a perfect crystal on a regular *k*-grid in reciprocal space using the primitive unit cell, a *k*-point aware, block-sparse treatment of the CC and EOM-CC equations is not yet available in the implementation employed in this work. Even for DFAs, it is well-known that the super-cell size convergence is an impractical approach for solving a proper *k*-point summation. The IP- and EA-EOM-CCSD method exhibit a computational scaling of  and ,^10^ respectively, where *N*_*o*_^unit^, *N*_*v*_^unit^ and *N*_*k*_ denote the number of occupied and unoccupied orbitals per unit cell and the number of *k*-points. The memory scaling is dominated by the *T̂*_2_-amplitudes and is proportional to . If instead of *N*_*k*_*k*-points, a super-cell approach with *N*_*u*_ = *N*_*k*_ unit cells is employed such that *N*_*o*_^super^ = *N*_*u*_*N*_*o*_^unit^ and *N*_*v*_^super^ = *N*_*u*_*N*_*v*_^unit^, a  and a  computational scaling for the IP- and EA-EOM-CCSD method, respectively, is the consequence. Analogously, the memory scaling becomes proportional to . Thus, a *k*-point based treatment of the EOM-CC equations would result in a reduced computational scaling of a factor *N*_*k*_^2^ and a reduction in memory scaling by a factor of *N*_*k*_. It must, however, be stressed that in accordance with Bloch’s theorem, the Born–von-Karman (BvK) cell of a *M* × *K* × *L* super cell evaluated at a single *k*-point ***k***_off_ is identical to the BvK cell resulting from a primitive unit cell being evaluated on a regular *M* × *K* × *L k*-grid shifted by ***k***_off_. Hence, even though the super-cell approach is notably more computationally expensive, the numerical EOM-CCSD results presented here are not affected by this choice. Still, it is well-known for standard electronic-structure theory, that achieving convergence in the super-cell approach quickly becomes intractable.

For the EOM-CC methods, we currently perform calculations of increasing system size and perform an extrapolation to the TDL,^10,22,23^ which requires knowledge about the convergence rate of the correlation energy with respect to the system size. While this convergence rate has been studied for the ground-state of the 3-dimensional case of a bulk solid,^20,24^ the formal convergence behavior for electronically excited states (charged or neutral) for any dimension is unknown.

In this work, we formally derive the analytical expression governing the convergence rate of the band gap on the IP/EA-EOM-CCSD level of theory. Subsequently, we verify the correctness of the derived expression by applying it to the band gap of a single chain of *trans*-polyacetylene, demonstrating an efficient extrapolation approach to the TDL. Furthermore, by repeating the calculations using the *G*_0_*W*_0_ method with a HF starting point (*G*_0_*W*_0_@HF), we show that the derived convergence rate is not specific to periodic EOM-CC theory but can be used for other correlated methods as well.

## 2 Theory

### 2.1 EOM-CC Theory

The EOM-CC framework is an extension to ground-state CC theory to compute properties of excited states. Depending on the nature of the excitations, different EOM-CC methods are available: The most prominent ones are EE-EOM-CC, for neutral electronic excitations, IP-EOM-CC for ionization processes and EA-EOM-CC for electron attachment processes. For the present work only the latter two methods are of importance, as the fundamental band gap of a material is given by the sum of its lowest ionization potential and its electron affinity.

The starting point of the EOM-CC method is the ground-state CC many-electron wave function |Ψ_0_⟩, which is defined by an exponential ansatz
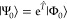
1with the Slater determinant |Φ_0_⟩, which is the ground-state wave function of a preceding mean-field calculation, usually HF.  is the so-called cluster operator, which can excite up to *M* electrons
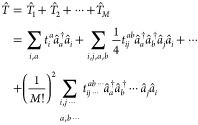
2with the coefficients *t*_*i*_^*a*^, *t*_*ij*_^*ab*^, ··· being again the cluster amplitudes and  and  the creation/annihilation operators in second quantization, creating/annihilating an electron in orbital *p*/*q*. For the sake of brevity, the notation in eq 2 is such that *i*, *j*, *k* denote occupied and *a*, *b*, *c* unoccupied spin orbitals. From Section 2.2 onward, we employ spatial orbital representation. We refer the reader to refs (25 and 26) for the full set expressions. If *M* is equal to the number of electrons *N* of the system, the ansatz in eq 1 is exact. For reasons of otherwise impractical computational scaling, *T̂* is truncated in practice. For example, M = 2 corresponds to CC theory with single and double excitations (CCSD), which is also used in this work.

To compute the wave function of the *n*-th excited state, the EOM-CC framework starts from a linear ansatz
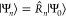
3where  is an excitation operator similar to the cluster operator *T̂*. For IP-EOM-CC and EA-EOM-CC,  assumes the form
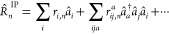
4a
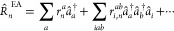
4b

The *r*-coefficients contain the description of the wave function of the *n*-th excited state and will be summarized under the excitation vectors |*R*_*n*_^IP^⟩ or |*R*_*n*_^EA^⟩. The objective of the EOM-CC methodology is to determine these coefficients. In analogy to the *T̂*-operator, the operators defined in eqs 4a and 4b contain all possible processes to excite an *N*-electron system into an (*N* – 1) electron state and an (*N* + 1) electron state, respectively. However, for the same reasons stated before for ground-state CC,  and  are truncated in practice. The most common approximation for EOM-CC involves only the first two terms of eqs 4a and 4b and is termed IP-EOM-CCSD and EA-EOM-CCSD, respectively. Consequently, IP-EOM-CCSD accounts only for 1-hole and 2-hole–1-particle-excitation processes, while EA-EOM-CCSD is restricted to 1-particle– and 2-particle–1-hole processes. We stress that, here, the term excitation process includes the removal or addition of an electron.

In order to determine the excited states |*R*_*n*_^IP/EA^⟩ and the related IP- or EA-energy, the eigenproblem
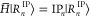
5a
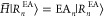
5bneeds to be solved, where  is the similarity-transformed Hamiltonian, with *T̂* being the ground-state CC cluster operator. Due to the generally intractable size of *H̅* in the chosen representations, the eigenvalues and vectors cannot be determined directly but need to be computed via an indirect approach like Davidson’s method.^27^

Once the excited states |*R*_*n*_^IP/EA^⟩ are obtained, the corresponding IPs or EAs can be computed via
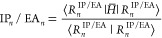
6

Note here, that even though *H̅* is nonsymmetric for the given *T̂* and therefore every eigenvalue is associated with both a left and a right eigenvector, the calculation of the eigenvalue in eq 6 only requires the knowledge of one of the eigenvectors. The working equations for  can be found in, e.g. ref (10).

Finally, note that while the theoretical framework of CC theory for the ground and excited states was elucidated using spin orbitals, henceforth all quantities will be expressed in terms of (spin-independent) spatial orbitals, as all the results shown in this work have been obtained without the consideration of spin degrees of freedom.

### 2.2 EOM-CC Structure Factor

Let us now introduce an expression for the IPs and EAs that makes it possible to analyze their dependence on the interelectronic distance. A closer look of the working equations show that all contributions to the expectation value in eq 6 constitute contractions of cluster amplitudes (see eq 2), *r*-coefficients (eq 4a/4b) and Coulomb integrals
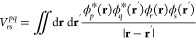
7where ϕ_*p*_(**r**) denotes a single-particle state of the underlying mean-field theory (HF in this work).

By assuming BvK boundary conditions we can introduce the codensities in reciprocal space
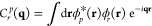
8

One can rewrite eq 7 as
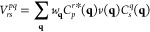
9

The discrete **q**-vectors in eqs 8 and 9 lie on a grid in reciprocal space and  is the Coulomb potential in reciprocal space for the three-dimensional case. We stress that the **q**-mesh is used to represent the codensities in Fourier space. If the single-particle states are expressed using Bloch’s theorem such that , where *u*_*s*_(**r**) is a cell periodic function and **k**_*s*_ is a wave vector in the first Brillouin zone, the **q**-vectors correspond to the difference between the corresponding wave vectors and a reciprocal lattice vector of the periodic unit cell. If the single-particle states are represented using a super-cell approach, the **q**-vectors correspond to reciprocal lattice vectors of the super cell. Both approaches are formally equivalent, although Bloch’s theorem enables a computationally more efficient implementation. *w*_**q**_ is a weighting factor that depends on the employed integration grid and method. It follows from eq 8 that *C*_*s*_^*q*^(0) is equivalent to the overlap between the two involved single particle states
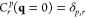
10

The terms contributing to the expectation value of IP- and EA-EOM-CCSD of eq 6, that is the IP and EA, can be broadly separated into two types, single-body and many-body contributions.

We define single-body mean-field contributions to explicitly depend on the Fock matrix elements *f*_*q*_^*p*^ and only contain Coulomb integrals implicitly by virtue of the Hartree and exchange contribution of the Fock matrix elements. A representative single-body mean-field contribution to the IP and EA is given by
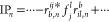
11aand
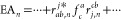
11b

We define IP_*n*_^(1)^/EA_*n*_^(1)^ as the sum of all single-body mean-field terms in the expression for IP_*n*_/EA_*n*_. It should be noted that the commutator expansion of the similarity transformed Hamiltonian also gives rise to effective single-body contributions originating from the contraction of the two-body Coulomb operator with different orders of  and . However, in this work we choose to include only terms from the underlying mean-field Hamiltonian in our definition of single-body terms.

**Figure 1:**
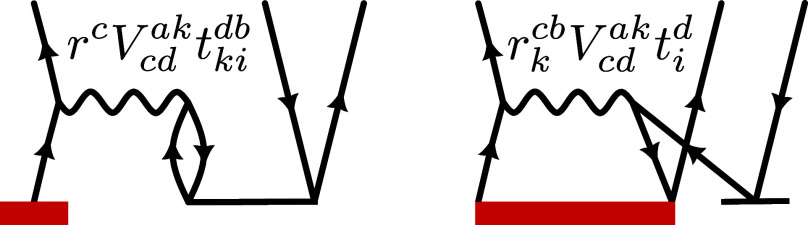
Goldstone diagrams and algebraic expressions for two exemplary many-body processes in the EA-EOM-CCSD equations.

It follows from our definition of IP_*n*_^(1)^/EA_*n*_^(1)^ that all remaining contributions to IP_*n*_/EA_*n*_ are referred to as many-body terms and depend explicitly on Coulomb integrals given by, for example
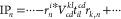
12aand
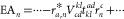
12bwhere *r*_*n*_^*i*^,*r*_*a*,*n*_ and *r*_*ij*,*n*_^*a*^,*r*_*i*,*n*_^*ab*^ denote the single- and double excitation coefficients of the *n*-th IP- and EA-EOM-CCSD excitation operator |*R*_*n*_^IP/EA^⟩, respectively. A diagrammatic representation of two exemplary many-body EA-EOM-CCSD contributions is shown in Figure 1.

By replacing all explicit occurrences of the Coulomb integrals *V*_*rs*_^*pq*^ in the many-body contributions by the decomposition in eq 9, factoring out *v*(**q**), and by contracting over all particle- and hole-indices involved in the evaluation of eq 6, one arrives at an expression for the IP_*n*_ and EA_*n*_ expectation value as a sum of IP_*n*_^(1)^/EA_*n*_^(1)^ and a product of the EOM-CC structure factor *S*_*n*_^IP/EA^(**q**) with the Coulomb potential *v*(**q**) as shown in eqs 13a and 13b
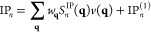
13a
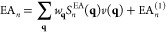
13bwhere the first term of eq 13a (eq 13b) contains all the many-body, or “correlation” contributions to the IP_*n*_ (EA_*n*_) as introduced in eq 12a, while the second term IP_*n*_^(1)^ (EA_*n*_^(1)^) denotes the sum of all single-body mean-field contributions defined in eq 11a.

The above expression gives access to the dependence of the “correlation” energy contribution to IP_*n*_/EA_*n*_ on the momentum transfer vector **q**, making it possible to perform a Fourier transform of *S*_*n*_^IP/EA^(**q**) and attribute contributions to IP/EA_*n*_ on the interelectronic distance. In this work, however, we restrict ourselves to studying the dependency on the momentum transfer vector **q**.

### 2.3 EOM-CC Structure Factor at **q** = 0

Since *C*_*s*_^*q*^(0) = δ_*q*,*s*_, the contributions to *S*_*n*_^IP/EA^(**q** = 0) can be further simplified. In particular, one discovers that *S*_*n*_^IP/EA^(**q** = 0) corresponds to the many-body character of the IP or EA state. We define the single-body character *p*_1_ of an IP or EA state as
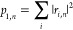
14aand
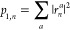
14brespectively, where the *r*-coefficients from eqs 4a and 4b are used. In this definition, the single-body character *p*_1,*n*_ of the *n*-th IP or EA state is given by the contribution of single-hole or single-particle processes to the overall description of the (*N* – 1) or (*N* + 1) electron wave function. For a normalized excited state |*R*_*n*_^IP/EA^⟩, *p*_1,*n*_ lies between 0 and 1. As we will show, for both IP- and EA-EOM-CCSD
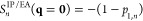
15holds. Thus, the value of the EOM-CC structure factor at **q** = **0** is equal to the negative many-body character of the excitation. This follows from eq 10, because only terms of the EOM-CCSD equations that depend on Coulomb integrals of type *V*_*cd*_^*ab*^, *V*_*kl*_^*ij*^, *V*_*bj*_^*ai*^ or *V*_*jb*_^*ia*^ contribute to the value of the EOM-CC structure factor at **q** = 0. All other terms give vanishing contributions to the structure factor at **q** = 0 due to the orthogonality of the orbitals, e.g. *C*_*i*_^*a*^(0) = ∫d**r**ϕ_a_^*^(**r**)ϕ_*i*_(**r**) = 0. That is, the integrals must be representable as products of hole–hole or particle–particle codensities following eq 9. In the case of IP-EOM-CCSD, there are 5 terms which contribute to *S*(**q** = 0), which are
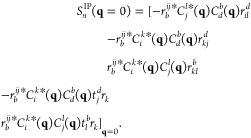
16

We skip the state index *n* in all EOM amplitudes *r* for brevity. By making use of the fact that at **q** = 0, the codensities reduce to overlap integrals between the single-particle states (see eq 10), eq 16 reduces to
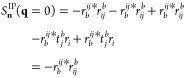
17which—for a normalized EOM-CCSD excitation vector—is equivalent to eq 15. The derivation for the EA-EOM-CCSD structure factor is analogous.

We emphasize that the value for *S*_*n*_^IP/EA^(**q** = 0) is a direct consequence of the chosen definition of IP_*n*_^(1)^/EA_*n*_^(1)^ and the eigenvalue in eq 6. Although this choice is helpful for the analysis of finite-size errors, there also exist alternative approaches, for example, using left eigenvectors as bra-states in eq 6, resulting in a different value for *p*_1,*n*_ and *S*_*n*_^IP/EA^(**q** = 0). We expect that for the systems studied in this work, which exhibit a very small many-body character, both approaches discussed above would yield very similar results.

The single-body character of the IP/EA-EOM-CCSD states has been used in previous studies to explain the accuracy of a variety of CC methods for molecular IPs^28−30^ and neutral double excitations.^31^ A reduced single-body character requires in general higher orders of cluster operators to achieve higher accuracy. Alternatively, a multiconfigurational approach could also be useful in such cases.^32^

### 2.4 Long-Range Behavior of the EOM-CC Structure Factor

To determine the asymptotic behavior of the EOM-CC structure factor in the long-wavelength limit, that is for |**q**| → 0, one can perform a Taylor series expansion around **q** = 0 by computing the derivatives of the EOM-CC structure factor with respect to **q**. The only explicit **q**-dependence of that quantity comes from the codensities *C*_*r*_^*p*^(**q**). As laid out in Section 2.2, *v*(**q**) is not included in the expression for *S*^IP/EA^(**q**), so that the products of codensities *C*_*r*_^p*^(**q**)*C*_*s*_^*q*^(**q**) are the only **q**-dependent quantities. We note that we neglect the implicit **q** dependence possibly introduced by the EOM-CC amplitudes (*r*_*i*_, *r*^*a*^, *r*_*ij*_^*a*^, *r*_*i*_^*ab*^). This assumed **q** independence of the *r*-amplitudes for **q** → 0 is justified by the fact that for systems with a band gap none of the quantities that appear in the EOM-CC equations, which are the Fock matrix elements, the Coulomb integrals and the ground-state *T̂*-amplitudes, depend on **q** (assuming the crystal momentum is conserved). While this is evident for the Fock matrix and the Coulomb integrals, the **q** independence of the *T̂*_2_-amplitudes is necessary for the ground-state transition structure factor to yield the correct 1/*N*_*k*_ convergence behavior in the thermodynamic limit (**q** → 0).^19^ For the *T̂*_1_-amplitudes, which do not explicitly appear in the expression of the ground-state CC correlation energy or the transition structure factor, a similar argument as for the EOM-CC equations applies: Since the *T̂*_1_-amplitudes are determined iteratively from **q** independent quantities, it is reasonable to assume that they themselves do not exhibit any dependence on **q** either.

By making use of the product rule and by realizing that a codensity at **q** = 0 is equivalent to the overlap between the two involved single particle states as defined by eq 10, one finds that
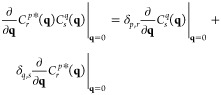
18

Equation 18 reveals, that if the codensities and by extension the Coulomb integrals in the EOM-CCSD equations would only couple holes with particles, the first derivative of the EOM-CC structure factor would vanish. In passing we note that this is the situation for the ground-state CC transition structure factor, which is why the lowest **q**-order in the long-wavelength limit for insulators is quadratic.^19^ The EOM-CC working equations, however, do also feature contractions with Coulomb integrals, which couple holes with holes and particles with particles, so that formally a linear contribution in **q** to the EOM-CC structure factor must be considered, because in general .

By repeating this procedure for the second derivative, one finds that the quadratic contribution does in general not vanish either, so that we can approximate the asymptotic behavior of the EOM-CC structure factor up to second order
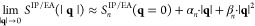
19where α_*n*_ and β_*n*_ are constants and *S*_*n*_^IP/EA^(**q** = 0) is the value of the EOM-CC structure factor at **q** = 0 as discussed in Section 2.3. We note that in eq 19, we assume *S*_*n*_^IP/EA^(**q**) to be spherically symmetric, which—while not generally the case—simplifies the following derivation and analysis.

Note that the above derivation—while based on some assumptions—did not depend in any way on the dimensionality of the system.

### 2.5 Convergence Rate of IPs, EAs and Band Gaps to the TDL

Based on the discussion from the previous sections, we now want to make a statement about the asymptotic convergence behavior of computed IPs, EAs and band gaps to the TDL. The TDL is approached for super cells with increasing size or increasingly dense *k*-mesh used to sample the first Brillouin zone. This corresponds to a continuous **q**-representation of the EOM-CC structure factor such that eqs 13a and 13b are given by
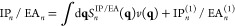
20

Finite-size errors of, for example, IP/EA energies are defined by the difference between calculations using finite system sizes and the TDL. For the above equation this corresponds to the difference between a continuous integration and a discrete sampling. Similar to the case of ground state CC calculations, we assume that the values of the EOM-CC structure factor at the sampled **q** points converge rapidly with respect to the employed system size, which will be later verified numerically. In other words, *S*_*n*_^IP/EA^(**q**) = *S*_*n*_^IP/EA-(TDL)^(**q**), where *S*_*n*_^IP/EA^(**q**) and *S*_*n*_^IP/EA-(TDL)^(**q**) refer to the structure factor from the finite system and in the TDL, respectively. Note that in this approach **q** is restricted to a discrete subset depending on the system size, whereas it is continuous in the TDL. In this case, the largest contribution to the finite-size error originates from the employed finite simulation cell size and the neglect of long-range interactions in real space corresponding to small **q**-vectors. Under these assumptions it is reasonable to define the following estimate for the finite-size error of the correlation contribution Δ_FS_^IP/EA^
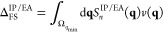
21

The integral in eq 21 is carried out over a sphere Ω_*q*_min__ centered at the Γ point with radius *q*_min_. The radius should be understood as a measure for the smallest reciprocal lattice vector of the considered simulation cell. For the 3-dimensional case, we evaluate the integral in eq 21 using the Fourier transform of the Coulomb potential in three dimensions which is
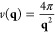
22so that—using *q* = |**q**|—a measure of the finite-size error is given by
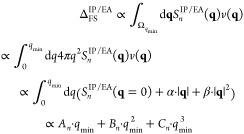
23where all constants resulting from the integration are collected in the parameters *A*_*n*_, *B*_*n*_ and *C*_*n*_. Note that the above equation is derived assuming spherical symmetry (first to second line) and inserting the truncated Taylor series from eq 19 (second to third line). The state index *n* of Δ_FS_^IP/EA^ has been omitted for brevity here and in all subsequent equations. By assuming spherical symmetry of the EOM-CC structure factor, the derivation in eq 23 is simplified substantially.

More practical is eq 23 if expressed as a function of the total number of **k**-points *N*_*k*_ of a uniform *k*-mesh. Noting, that
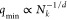
24where *d* is the dimension of the system. By applying this equality for the three-dimensional case, eq 23 becomes
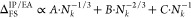
25

#### 2.5.1 Convergence Rates for Low Dimensional Systems

Since we assume that the asymptotic behavior of the EOM-CC structure factor is independent of the dimension of the system, we can now make use of eq 19 to derive the leading-order rate of convergence to the TDL for one- and two-dimensional systems. To this end we make use of the Fourier transforms of the Coulomb potential in the two- and one-dimensional case^33^
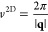
26and
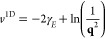
27respectively, where γ_*E*_ denotes the Euler constant. Note that we employ the lower dimensional FT of the Coulomb potential here even though realistic low-dimensional systems generally have a finite extent in the directions perpendicular to the a one or two dimensional material. In the long-wavelength limit, however, which is the focus of the present work, these contributions become negligible in comparison to those of the sheet or chain direction(s). This justifies the representation of the Coulomb potential by its lower-dimensional Fourier transforms. By repeating the same steps as for the derivation of the 3-dimensional convergence rate, we find that the finite-size error in a two-dimensional system converges like
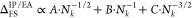
28and for a one-dimensional system the convergence rate is given by
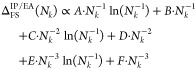
29

A summary of the derived convergence rates for 1, 2, and 3 dimensions is given in Table 1. We reiterate that these convergence rates are estimated from the contributions to IP_*n*_/EA_*n*_ around **q** = 0 in a sphere with a radius decreasing as *N*_*k*_ increases. It should also be noted that the actual convergence rate of numerically computed IPs/EAs depends on the chosen treatment of the Coulomb singularity in the respective computer implementation, which will be discussed in the following section.

**Table 1:** Convergence Rates for One-, Two- and Three-Dimensional Systems up to Second Order

dimension	convergence rate as a function of *N*_*k*_
1D	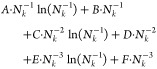
2D	*A*·*N*_*k*_^–1/2^ + *B*·*N*_*k*_^–1^ + *C*·*N*_*k*_^–3/2^
3D	*A*·*N*_*k*_^–1/3^ + *B*·*N*_*k*_^–2/3^ + *C*·*N*_*k*_^–1^

### 2.6 Convergence Rates and Treatment of Coulomb Singularity

The convergence rates derived in the previous section assume a spherically truncated integration around **q** = 0 to estimate the finite-size errors Δ_FS_^IP/EA^. In practical ab initio calculations, however, there exist a variety of treatments to approximate the integral around the Coulomb singularity at **q** = 0, which strongly influence the convergence rates as can already be observed for the exchange energy contribution.^34−39^ We now briefly discuss the significance of the treatment of the Coulomb singularity for the convergence rates derived in the previous section.

For the present study, we employ a Coulomb singularity treatment that captures the contribution of the *S*_*n*_^IP/EA^(**q** = 0) term to the integral in eq 23 exactly if the value of *S*_*n*_^IP/EA^(**q** = 0) is converged with respect to system size. As a consequence, the contributions to the finite-size errors proportional to *A* given in Table 1 will already be accounted for and the expected next-to-leading order contribution to the finite-size error will be proportional to *B*. In particular, the plane wave basis set calculations of the present work compute the average Coulomb kernel for the volume element at **q** = 0 to estimate its contribution to the integral.^38^

We note that there also exist other approaches in the literature that disregard the Coulomb singularity contribution and obtain EOM-CC band gaps by extrapolation. References (10, 11, and 22) disregard the **q** = 0 contribution already in the underlying HF calculation. As a consequence the HF band gap is underestimated and converges only as *N*_*k*_^–1/3^ in three-dimensional systems. The same applies to the finite-size error in EOM-CC theory as shown in the previous section. It should be noted that the finite-size errors from HF and EOM-CC partly cancel each other. However, the extrapolation procedure still requires a careful checking and can lead to errors that are difficult to control due to next-to-leading order contributions to the finite-size errors.

## 3 Computational Details

The numerical results presented in this work have been obtained using three different software packages. All EOM-CC calculations were performed using the CC4S software,^20,40^ which has been interfaced to FHI-aims^41,42^ and the Vienna Ab Initio Simulation Package (VASP).^43,44^ FHI-aims and VASP employ a numeric atomic orbital and plane wave basis, respectively. These basis sets correspond to a different basis representation of the matrix elements employed by CC4S, where the working equations of the IP- and EA-EOM-CCSD methods and their respective structure factors were implemented. As the representation of the EOM-CC structure factor requires the utilization of a plane wave basis, the interface to VASP was used to compute the structure factors for the IP- and EA-EOM-CCSD. The IPs and EAs can be computed using both interfaces. Furthermore, we employ FHI-aims and VASP for mean-field and *G*_0_*W*_0_ calculations.

For the VASP calculations the following settings were employed. In the case of the LiH EOM-CC and ground-state CC structure factors, which are discussed in Section 4.1, a relatively small basis set with 3 virtual closed-shell orbitals per occupied orbital was employed in order to compute the huge super-cell sizes necessary to resolve the structure factor for very small momentum vectors. Since we are concerned with the qualitative long-range description of the EOM-CCSD structure factor a small plane-wave basis set is sufficient. Similarly, the EOM-CC structure factors for *trans*-polyacetylene (tPA) were computed using a reduced basis set of 4 virtual closed-shell orbitals per occupied orbital. Due to substantial amount of vacuum in the simulation cell of the tPA chain, it is not practical to converge the results with respect to the plane-wave energy cutoff, which is why VASP was only used to obtain the EOM-CC structure factor but not the electronic band gaps themselves. Furthermore due to the computational overhead associated with additional vacuum when using a PW basis, the vacuum around the tPA chain was reduced to 7 Å. Specifically for the LiH calculations, the Li_GW and H_GW POTCAR files were employed, using a plane-wave energy cutoff of 300 eV. The tPA EOM-CCSD structure factor was computed with the C_GW_new and H_GW POTCAR files and an energy cutoff of 300 eV. To alleviate convergence problems of the HF and the CC calculations associated with the discretization of the Brillouin zone (BZ) for the highly anisotropic simulation cells of tPA, we employ the recently developed improved sampling method of the Coulomb potential in VASP.^38^

The following settings were used for the FHI-aims calculations. To determine the convergence to the TDL, the unit cell of a single tPA chain with 2 carbon atoms and 2 hydrogen atoms with at least 80 Å of vacuum in each direction perpendicular to the chain was optimized employing the B3LYP exchange–correlation functional,^45,46^ since this functional was shown in previous studies^47^ to reasonably reproduce the bond length alternation of tPA observed in experiment.^48^ For the geometry optimization a *tight* tier-2 basis set and a 1 × 1 × 20 **k**-grid was used. The CC and *GW* calculations involving FHI-aims were performed using the loc-NAO-VCC-*n*Z basis sets developed by Zhang et al.^49,50^ The basis set convergence for the band gap of tPA was investigated on the EOM-CCSD level of theory, the results of which are shown in Table 2. As Table 2 conveys, the loc-NAO-VCC-2Z basis set allows to converge the band gap to 157 meV for a 1 × 1 × 6 **k**-mesh and 141 meV for a 1 × 1 × 8 **k**-mesh, essentially independently of the size of the BvK cell, which we consider to be sufficient for the present application as the finite-size error rather than the basis-set incompleteness error is at the center of this study. Hence, all calculations involving FHI-aims will be performed using the loc-NAO-VCC-2Z basis set. In order to approximate the contribution from the singularity of the Coulomb potential, a variation of the spherical truncation approach pioneered by Spencer and Alavi^35^ is employed in FHI-aims. In this *cut-Coulomb* approximation the long-ranged part of the Coulomb potential is made to decay fast to 0 beyond a set radius *r*_cut_ by multiplying the original  potential with a complementary error function^51^
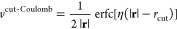
30with η as the inverse decay width.

**Table 2:** Basis Set Convergence of the tPA Band Gap Using the loc-NAO-VCC-*n*Z (*n* = 2, 3, 4) Basis Set with a 1 × 1 × 6 and a 1 × 1 × 8 **k**-mesh^a^

	2Z	3Z	4Z
*E*_gap_^1×1×6^	5.859	5.765	5.702
*E*_gap_^1×1×8^	5.403	5.318	5.262

a Energies are given in units of eV.

## 4 Results

To determine the validity of the convergence rate of the finite-size error for IP and EA-EOM-CCSD, the findings will be presented as follows: first, the fundamental properties of the EOM-CC structure factor that have been mentioned in part already in Section 2, will be elucidated by looking at the structure factor of the 3D LiH primitive cell repeated periodically in one direction. Following that qualitative discussion, the previously derived convergence rates of the EOM-CC band gap finite-size error will be applied to a one-dimensional system, a chain of *trans*-polyacetylene. Finally, the analytical extrapolation expression will be applied to the finite-size convergence of the *G*_0_*W*_0_ band gap, testing its validity outside of EOM-CC theory.

### 4.1 EOM-CC Structure Factor of LiH in One Dimension

In order to relate the herein newly derived EOM-CC structure factor to the ground-state analogue, the *transition structure factor*,^19,20^ we want to start by computing these two quantities for the LiH chain using super cells, which consist of increasingly many unit cells in a single direction. Since the purpose of this section is a direct comparison of the correlation structure factors of the two methods, only the Γ-point was sampled in reciprocal space. As noted in Section 2.4, the derived behavior of *S*^IP/EA^(*q*) for *q* → 0 (and equally so for the transition structure factor) should hold independently of the system’s dimension.

**Figure 2:**
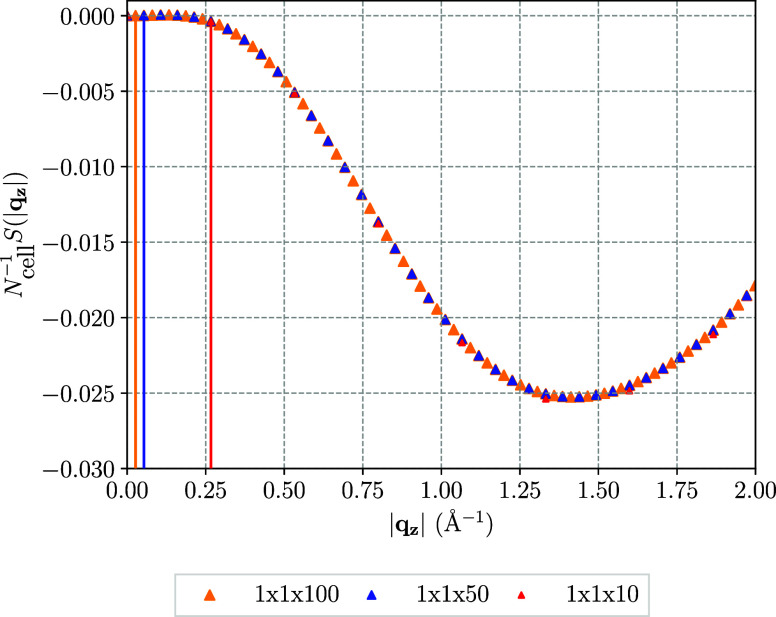
Ground state CCSD transition structure factor of LiH plotted along the *z*-axis using a 1 × 1 × 10, 1 × 1 × 50 and a 1 × 1 × 100 super cell extended in one dimension. The vertical lines mark the minimum distance between any two lattice points in the reciprocal lattice. To make the transition structure factor of different BvK cell sizes comparable, the structure factor is scaled by one over the number of unit cells in the respective super cell, *N*_cell_^–1^.

We shall start by reviewing the properties of the ground-state CCSD transition structure factor, which is shown in Figure 2 for the LiH chain for super-cell sizes of 1 × 1 × 10, 1 × 1 × 50 and 1 × 1 × 100. Subsequently, a comparison to the EOM-CC structure factor will be drawn.

In analogy to the EOM-CC structure factor, which upon contraction with the Coulomb potential and integration over the reciprocal space yields the correlation contribution to the charged quasiparticle energies (see eq 20), the ground-state correlation energy *E*_0_^corr^ can be obtained in the same way by means of the transition structure factor *S*(**q**)
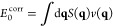
31

Figure 2 shows the medium to long-range portion of the transition structure factor in the direction of the chain, that is the *z*-direction. Even though, *S*(**q**) can be computed for longer **q**-vectors, corresponding to short-range processes in real space, this is not relevant to the present discussion of the finite-size convergence. The vertical lines in Figure 2 show the magnitude of the smallest **q**-vector that can be resolved in the respective super cell. That minimal **q**-vector is given by the minimal distance of two **k**-points or (in a super-cell formulation) by the smallest reciprocal lattice vector and corresponds to the biggest real-space distance captured by the BvK cell. As can be observed in Figure 2, the bigger the super-cell size of LiH, the smaller does this minimal **q**-vector become and the more long-range correlation information does the transition structure factor contain. The transition structure factor in Figure 2 features a minimum, at some material and state-specific distance in reciprocal space. This distance can be interpreted as a characteristic distance for the electronic correlation, as we expect the contribution of the CCSD transition structure factor to the correlation energy to be maximal in the vicinity of its extremum. In the specific case of the LiH chain, one finds that the minimum is at 1.4 Å^–1^ for the ground state correlation energy, which corresponds to approximately a 1 × 1 × 2 super cell. Another fundamental property of the transition structure factor of the ground-state is the fact that for |**q**| → 0, *S*(**q**) → 0 with zero slope as is apparent in Figure 2. This directly results from the sum-rule of the pair correlation function, which is the Fourier transform of the transition structure factor.

**Figure 3:**
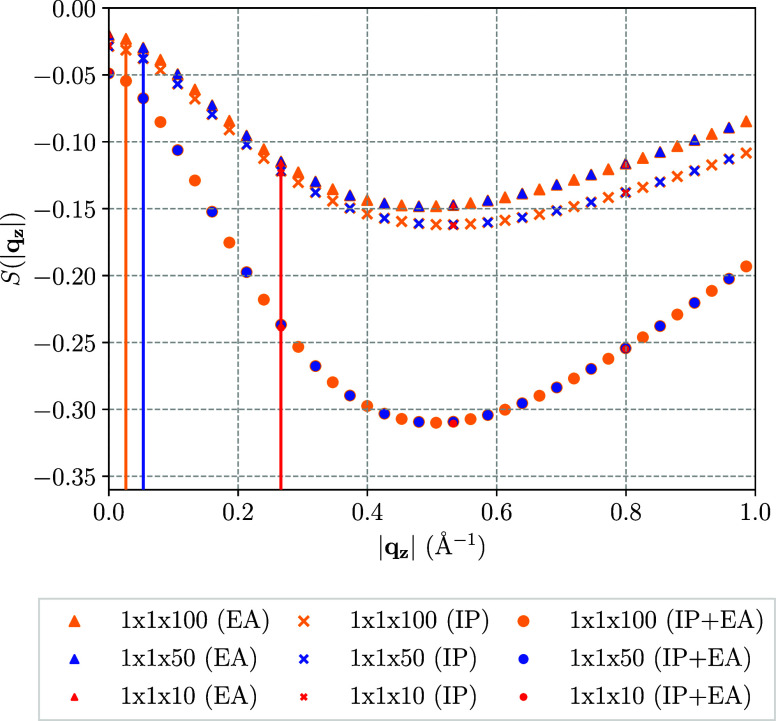
IP-EOM-CCSD, EA-EOM-CCSD transition structure factors and the sum of both corresponding to the band gap of LiH plotted along the *z*-axis using a 1 × 1 × 10, 1 × 1 × 50 and a 1 × 1 × 100 super cell extended in one dimension. The vertical lines mark the minimum distance between any two lattice points in the reciprocal lattice.

The EOM-CC structure factor for charged excitations exhibits qualitative differences to the ground-state transition structure factor. The structure factor for the first IP and EA in the EOM-CCSD framework for the LiH chain is shown in Figure 3.

The EOM-CC structure factors feature a minimum as well, but it appears at a significantly smaller |**q**| value of 0.5 Å^–1^, which corresponds approximately to a 1 × 1 × 6 super cell. This reflects the longer range of correlation effects in the EOM-CC case in comparison to the ground-state. This comparison clarifies that the problem of reaching the TDL for charged quasiparticle energies is substantially more difficult than for the ground state case. Another point of departure between ground state CC and EOM-CC theory, is the value of the structure factor at **q** = 0. While in the case of the ground state the *S*(**q** = 0) value is always 0, in the case of EOM-CC we have previously derived that *S*(**q** = 0) is a finite negative value, in our formulation corresponding to the many-body character of the respective excitation.

As Figure 3 demonstrates, all the properties discussed for the EOM-CC structure factor apply in a similar fashion to both the IP and the EA-EOM-CCSD case and the IP + EA case. Let us stress once more that the IP + EA case corresponds to the electronic band gap in the presently used convention. It should be stressed at this point, that the EOM-CC structure factor that we have introduced in this work only captures the correlation contribution to the IP and EA quasiparticle energies and to the band gap, which are given by the many-body contributions of the EOM-CC equations as discussed in Section 2.2. In order to obtain the full excitation energy, we compute and converge the generally sizable single-body contributions (compare eq 11a) to the EOM-CCSD expectation value separately.

With respect to the previously derived asymptotic behavior of the EOM-CC structure factor for **q** → 0, it becomes immediately apparent from the LiH EOM-CC structure factors, particularly for the 1 × 1 × 100 cell, that it does not seem to exhibit a significant linear behavior at **q** = 0. Furthermore, *S*(**q** = 0) is relatively small. Therefore, one can conclude for the anisotropic LiH super cell, that both the constant |**q**|^0^ and the linear contribution |**q**|^1^ contribution of the EOM-CC structure factor to Δ_FS_^IP/EA^ is negligible.

### 4.2 *trans*-Polyacetylene Chain

#### 4.2.1 EOM-CC Structure Factor of tPA

**Figure 4:**
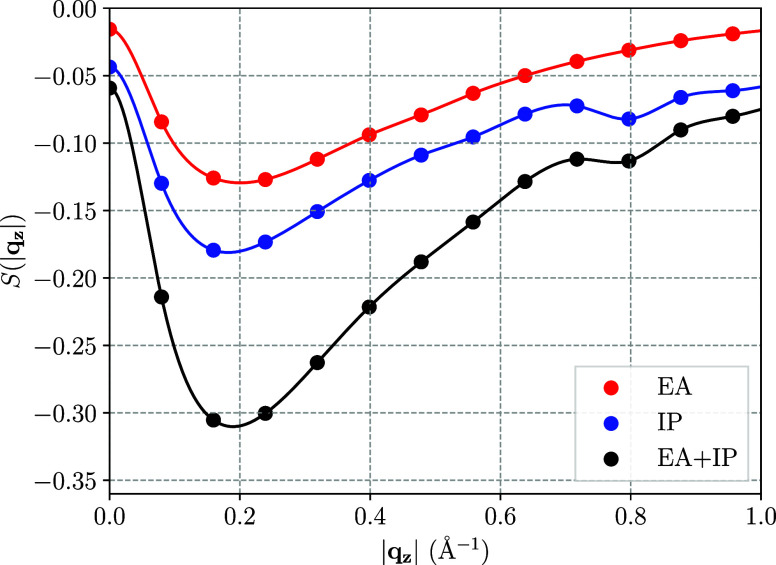
IP and EA-EOM-CCSD structure factor and their sum corresponding to the band gap of tPA along the direction of the chain for a 1 × 1 × 32 super cell (*z*-direction). As a guide to the eye, the calculated data points are connected via a cubic spline interpolation.

In order to verify the applicability of the convergence rates, which were derived in the long-wavelength limit (|**q**| → 0), we now investigate the IP and EA-EOM-CCSD structure factors of an actual one-dimensional system, the tPA chain (note, that the previously studied LiH system, is a bulk material, however, the super cell only extended in one direction). For that purpose, super-cell sizes of up to 1 × 1 × 32 were computed via IP and EA-EOM-CCSD. The IP and EA-EOM-CCSD structure factors, and the structure factor corresponding to the correlation energy contribution to the band gap, that is the sum of IP and EA in the present convention, are illustrated in Figure 4.

Figure 4 shows that for the 1 × 1 × 32 super cell of the tPA chain, the minimum of the EOM-CC structure factor is resolved and two data points to the left of the minimum are obtained. As discussed previously for the case of the LiH EOM-CC structure factor, the analytical extrapolation expressions derived for the asymptotic limit (|**q** → 0|) are strictly speaking only applicable for system sizes for which the minimum of the structure factor can be resolved. By applying a cubic spline interpolation to the EOM-CC structure factors in Figure 4, one finds that the minimum is located near 0.2 Å^–1^, which corresponds to approximately a 1 × 1 × 13 BvK cell. It is, however, important to note that the data point directly to the right of the minimum in Figure 4, which corresponds to a 1 × 1 × 10 BvK cell, is in close proximity to the minimum itself, so that the quadrature error resulting from extrapolation of system sizes as small as 10 primitive cells in one direction can be assumed to be small. Compared to the characteristic distance of 0.5 Å^–1^ previously found for LiH, this suggests that the length scale of the electronic correlation is more than doubled compared to LiH. One possible explanation for that is the presence of long-ranged dispersion interactions, which are expected to be more prominent in an organic compound like tPA than in an ionic compound with small ions like LiH. Note, that in the case of the IP-EOM-CCSD structure factor in Figure 4, we observe a second, shallow minimum at 0.8 Å^–1^. Since this feature is located in the medium to short-range region of the EOM-CC structure factor, this is most certainly an artifact of the comparably small PW basis set, which had to be used to compute the 1 × 1 × 32 super cell of tPA. However, as we are interested in the long-range characteristics of the electronic correlation, this is not expected to have any effect on the properties of the tPA EOM-CCSD structure factors discussed so far.

#### 4.2.2 Convergence of the Correlation Contribution

We study the convergence of the numerically determined band gap for *trans*-polyacetylene (*t*PA) to verify the derived convergence rate in eq 29 of one-dimensional systems to the TDL.

**Figure 5:**
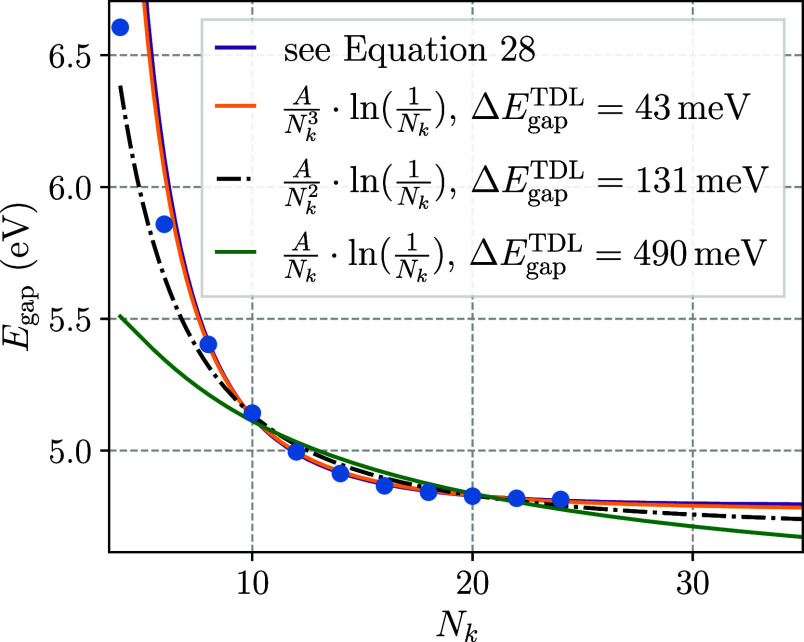
Application of the derived convergence model and its subsets to the convergence of a tPA chain’s energy band gap. The models were fitted to data points with *N*_*k*_ ≥ 8, where *N*_*k*_ denotes the total number of *k*-points used in the calculation. For the reduced models the deviation of the extrapolated band gap Δ*E*_gap_^TDL^ from the full model (purple) is shown.

**Figure 6:**
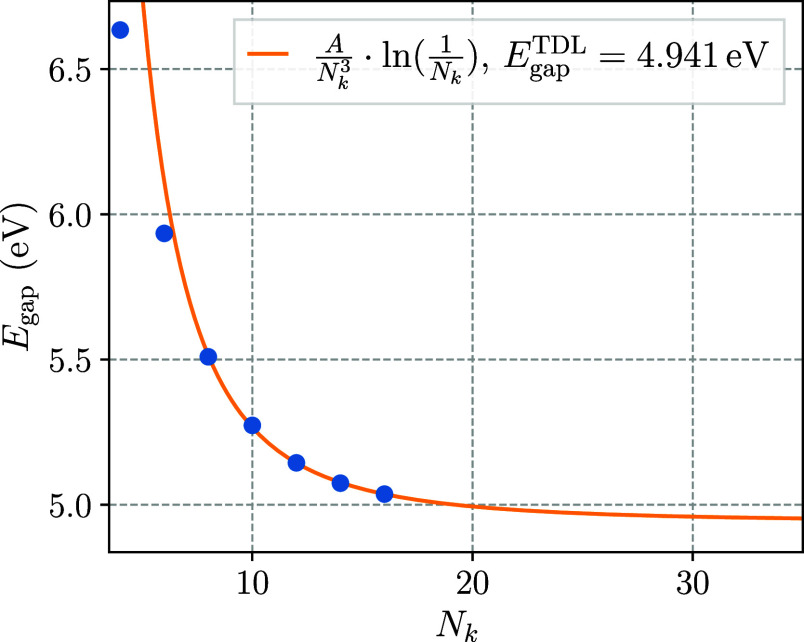
Application of the *AN*_*k*_^–3^ ln(*N*_*k*_^–1^) model to the convergence of the EOM-CCSD band gaps for the tPA3 chain. The model was fitted to data with *N*_*k*_ ≥ 8.

**Figure 7:**
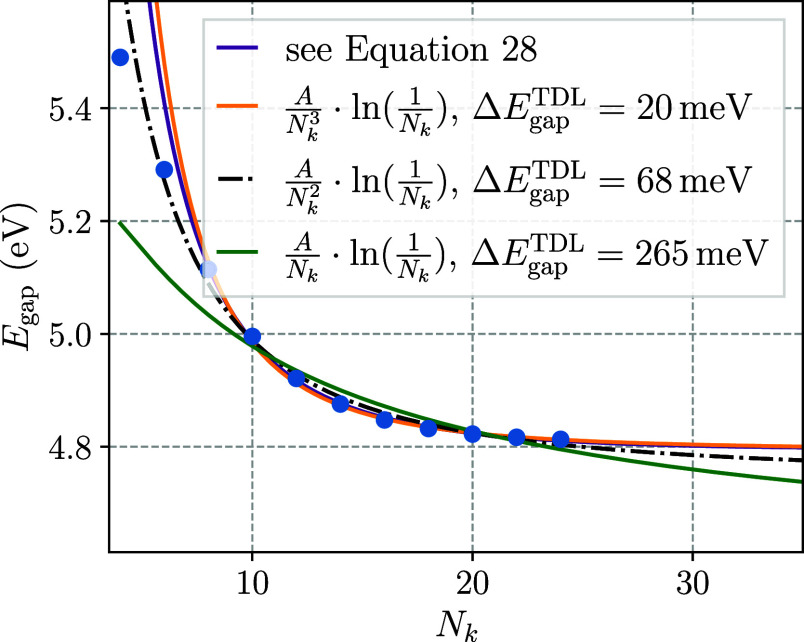
Application of the derived convergence model and its subsets to the convergence of the downsampled EOM-CCSD band gaps for a tPA chain. The models were fitted to data with *N*_*k*_ ≥ 8. For the reduced models the deviation of the extrapolated band gap Δ*E*_gap_^TDL^ from the full model (purple) is shown.

**Figure 8:**
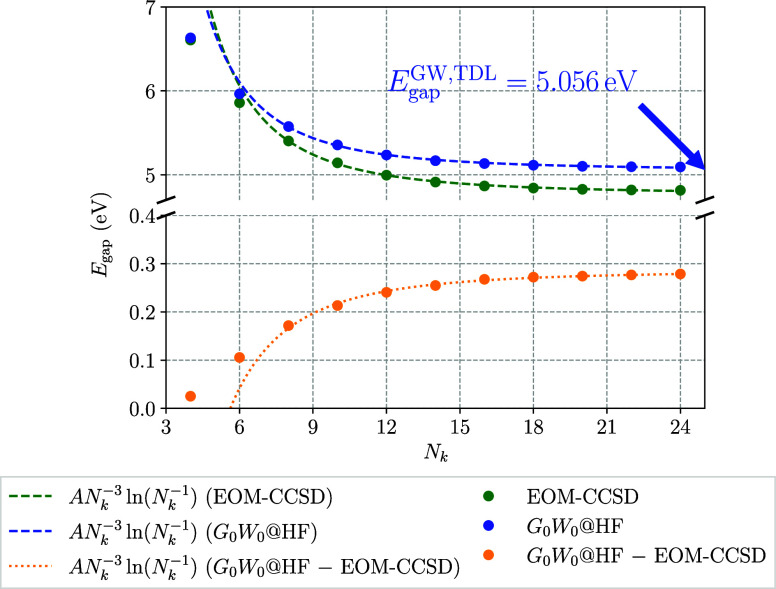
Comparison of the convergence to the TDL for IP/EA-EOM-CCSD theory and the *G*_0_*W*_0_@HF method for a single chain of tPA. The band gaps of both methods are modeled via an *AN*_*k*_^–3^ ln(*N*_*k*_^–1^) term, as they were shown to dictate the convergence to the TDL for EOM-CC in Section 4.2. The models were fitted to data with *N*_*k*_ ≥ 8.

**Figure 9:**
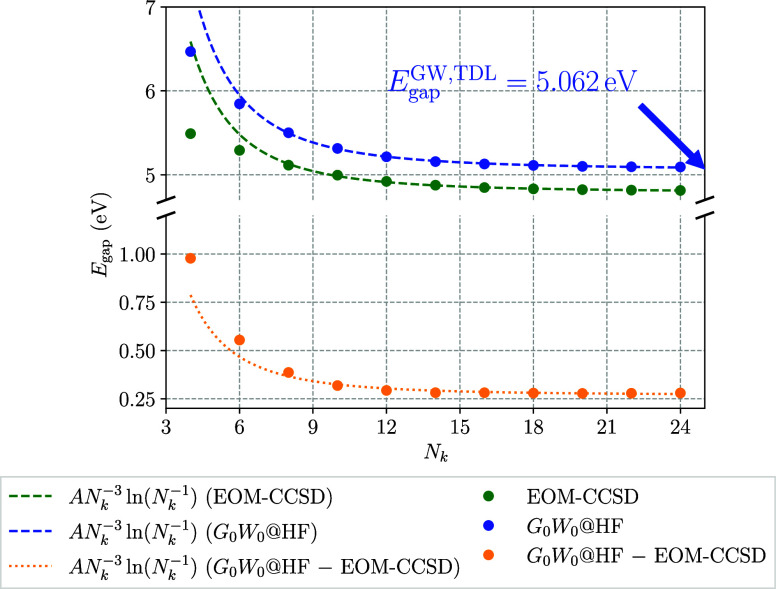
Comparison of the convergence to the TDL for IP/EA-EOM-CCSD theory and the *G*_0_*W*_0_@HF method for a single chain of tPA after applying downsampling. The band gaps of both methods are modeled via an *AN*_*k*_^–3^ ln(*N*_*k*_^–1^) term, as they were shown to dictate the convergence to the TDL for EOM-CC in Section 4.2. The models were fitted to data with *N*_*k*_ ≥ 8.

In the pursuit to determine if some order of *q* in *S*(*q*) of eq 19 is dominant and if the derived convergence rate in eq 29 can be reduced meaningfully to a single leading-order contribution, a set of convergence rate models are applied to the finite-size convergence of tPA in Figure 5. In particular, the full derived model in eq 29 is fitted to the calculated band gaps as a function of *N*_*k*_ along the direction of the tPA chain. This model is compared to three other convergence rates, which are the leading-order contributions originating from the *q*^0^, *q*^1^ and *q*^2^ contribution to the long-wavelength limit of the EOM-CC structure factor, which are *AN*_*k*_^–1^ ln(*N*_*k*_^–1^), *AN*_*k*_^–2^ ln(*N*_*k*_^–1^), and *AN*_*k*_^–3^ ln(*N*_*k*_^–1^), respectively, where *A* is determined by fitting to the numerical data. To prioritize the accurate description of the finite-size convergence in the long-range limit, a modified least-squares cost function was used for fitting, where a data point corresponding to a system size of *N*_*k*_ was weighted with a factor of *N*_*k*_^2^. In agreement with the computed EOM-CC structure factor of tPA shown in Figure 4, it was found that the derived convergence rates only describe the numerical data in Figure 5 qualitatively well for system sizes that exceed some minimal number of **k**-points *N*_*k*_, so that the models in Figure 5 are fitted to the EOM-CCSD band gaps for *N*_*k*_ ≥ 8. To remain consistent, all following fits to FHI-aims band gaps, that is Figures 6–9, are shown for *N*_*k*_ ≥ 4, but only fitted to data points with *N*_*k*_ ≥ 8.

Undeniably, the *q*^0^-order contribution to eq 29 on its own, given by *AN*_*k*_^–1^ ln *N*_*k*_^–1^, fails entirely to model the computed data in Figure 5, underestimating the band gap in the TDL by almost 500 meV relative to the full model. This lends credence to the aforementioned conjecture that *q*^0^ contributions would only play a minor role in the description of the convergence to the TDL for quasiparticle excitation with minor many-body character. It is also consistent with the small contribution of the |**q**|^0^ term of the EOM-CC structure factor to the correlation energy in the anisotropic LiH cell in Section 4.1. As a matter of fact, the many-body contribution of the IP and the EA in tPA were found to be both about 5%. Moreover, we stress that even if this contribution were large, the employed Coulomb singularity treatment in FHI-aims would capture this contribution to the finite-size error. Similarly, the model corresponding to a EOM-CC structure factor linear in **q**, given by *AN*_*k*_^–2^ ln(*N*_*k*_^–1^), converges visibly slower than the calculated data points leading to an underestimate of the band gap of over 130 meV compared to the full model. This, in combination with the observation that the LiH EOM-CC structure factor does not seem to exhibit any linear behavior for *q* → 0, hints at the possibility that in some systems—or possibly in general—there is no or only a negligible linear contribution to *S*^IP/EA^(**q**) for *q* → 0. Instead, at least in the case of tPA, the *q*^2^ contribution to the EOM-CC structure factor appears to dictate the TDL convergence of the band gap. Even though the *AN*_*k*_^–3^ ln(*N*^–1^) model notably deviates from the calculated data for smaller *N*_*k*_, it matches perfectly for *N*_*k*_ = 8 to *N*_*k*_ = 24, lying virtually on top of the full model and yielding a band gap underestimated by 43 meV compared to the full model.

The range of applicability is slightly larger than the prior investigation of the EOM-CC structure factors for tPA would suggest, were a minimum of 10 primitive cells or **k**-points in one direction were found to be necessary. One likely explanation for that minor disagreement is the utilization of different Coulomb potential approximations in both codes as detailed in Section 3.

To conclusively verify the precision and accuracy of both the EOM-CCSD band gap data obtained via FHI-aims and the herein proposed extrapolation approach, the EOM-CCSD band gap calculations for super cells of size up to 1 × 1 × 16 were repeated using one of the structures investigated by Windom et al.^52^ In that study, the fundamental band gap of different tPA geometries was investigated on the EOM-CCSD level of theory employing increasingly long tPA oligomers with a cc-pVTZ basis set. For the comparison, the central C_2_H_2_ unit of one of these structures, in the original paper denoted by tPA3, was extracted and treated under periodic boundary conditions in the same manner as the B3LYP-optimized structure studied so far. To ensure a fair comparison, the FHI-aims calculations were performed employing the loc-NAO-VCC-3Z basis. The results are shown in Figure 6.

Using the previously identified *AN*_*k*_^–3^ ln(*N*_*k*_^–1^) leading-order model, an EOM-CCSD band gap of 4.941 eV in the bulk-limit is found. This is in reasonable agreement with the estimated TDL value of 5.07 eV of the original study. The remaining deviation of roughly 130 meV is likely the result of comparably small oligomer sizes, namely 6 to 9 C_2_H_2_ units that were used for the extrapolation. Also, the fact that neither of the results are converged with respect to the basis set (see Table 2) makes an exact comparison more difficult.

#### 4.2.3 Convergence of the Mean-Field Contributions

What has been neglected so far, however, is the potential influence of the single-body contributions from the underlying HF calculations on the overall convergence of the band gap. Most importantly, the convergence of the HF exchange with respect to system size needs to be considered. So far, the HF calculations have been performed with the same system sizes as were subsequently used for the CC calculations, so that the total finite-size error of the final EOM-CCSD band gap contained contributions from both the mean-field calculation and the correlated CC calculation. To allow for a more systematic study, which only includes long-range correlation effects, the EOM-CCSD band gap results will be recomputed using converged HF single-particle states and eigenenergies from super cells of size 1 × 1 × 48 or more. Via this downsampling approach, one can independently analyze the convergence of the correlation energy of the EOM-CCSD band gaps. The finite-size convergence including the leading-order fits as they have been shown in Figure 5 is presented in Figure 7 for the downsampled data.

The fitted leading-order models in Figure 7 suggest that after the single-body contributions, particularly the HF exchange, has been accounted for, still the *AN*_*k*_^–3^ ln(*N*_*k*_^–1^) model which results from the contribution to the structure factor quadratic in *q*, describes the convergence to the thermodynamic limit best and virtually lies on top of the data points for *N*_*k*_ > 10.

### 4.3 Comparison to *G*_0_*W*_0_

To test the validity of the derived convergence rate for one-dimensional systems outside of CC theory, the leading-order contribution determined in the previous section is applied to the *GW* approximation, as well. For that purpose, for the same system sizes that were previously computed via IP and EA-EOM-CCSD, the band gaps were obtained using the *G*_0_*W*_0_ method with a HF starting point (*G*_0_*W*_0_@HF) to allow for a direct comparison to the EOM-CC results.

The performance of the previously extracted *AN*_*k*_^–3^ ln(*N*_*k*_^–1^) convergence rate applied to both EOM-CCSD and *G*_0_*W*_0_@HF is shown in Figure 8. There, one observes that the convergence behavior of the *GW* method with respect to the number of **k**-points *N*_*k*_ almost mirrors the one of the EOM-CCSD method. In the same way the leading-order term originating from the *q*^2^ contribution to the EOM-CC structure factor models the band gap convergence of both theories accurately over the entire range of *N*_*k*_ = 8 to *N*_*k*_ = 24.

In an analogous step as in Section 4.2, the comparison between EOM-CCSD and *G*_0_*W*_0_ band gaps was repeated after converging the underlying HF orbitals and eigenenergies via downsampling. As previously, for that purpose the HF calculation was performed on a **k**-grid of size 1 × 1 × 48 or more and the resulting HF orbitals and eigenenergies were subsequently used to compute the IP/EA-EOM-CCSD and *G*_0_*W*_0_ band gaps on **k**-meshes between 1 × 1 × 8 and 1 × 1 × 24. The resulting convergence to the bulk-limit for the two methods is shown in Figure 9.

Figure 9 shows that after the convergence of the single-body contributions of the underlying HF method has been ensured, the remaining contributions to both the EOM-CCSD and *G*_0_*W*_0_ quasiparticle energies converge with the previously identified leading-order model *AN*_*k*_^–3^ ln(*N*_*k*_^–1^). While the downsampling reduces the individual band gaps of both methods most notably for small values of *N*_*k*_, the convergence behavior and the band gap in the TDL remain unaffected, while the extrapolated *G*_0_*W*_0_@HF band gap in the TDL changes by less than 6 meV.

Figures 8 and 9 also show the differences between *G*_0_*W*_0_ and EOM-CCSD band gaps retrieved as a function of *N*_*k*_ without and with applying the downsampling technique, respectively. We note that the convergence of the difference can also be well approximated using an *AN*_*k*_^–3^ ln(*N*_*k*_^–1^) model. The relatively small magnitude of the fitting parameter indicates that both methods capture contributions to the correlation energy with a similar magnitude and the same leading-order behavior. In other words, although the *G*_0_*W*_0_ and EOM-CCSD band gaps exhibit a finite-size error that is similar in magnitude and converges with the same analytical behavior to the TDL, there are different contributions of the diagrams from both methods, which lead to small differences in the leading-order convergence prefactor. In practice one can still benefit from the similarity of both methods by correcting the finite-size error of EOM-CCSD band gaps using the computationally significantly cheaper *G*_0_*W*_0_ method. As noted before, the long-range behavior of the EOM-CC structure factor is independent of the dimension of the system. Therefore, one can infer that a *GW*-aided finite-size correction technique for EOM-CC theory can in principle be performed for one, two, and three-dimensional systems alike.

An important question remaining is: what diagrammatic contributions account for the differences between *G*_0_*W*_0_ and EOM-CCSD in the long-wavelength limit? Although, the present work does not provide an explicit answer, we note that previous studies performed detailed investigations of the relationship between the *GW* approximation and the EOM-CC framework. In the first study of this sort, performed by Lange and Berkelbach,^12^ it was found that the *G*_0_*W*_0_@HF approximation features more higher-order ring diagrams than the Green’s function of IP and EA-EOM-CCSD. These diagrams are known to be particularly relevant for the description of electronic correlation in the long-wavelength limit of the ground state for metallic systems.^53,54^ Since then, different flavors of the *GW* approximation have been reformulated in a CC-like fashion. Bintrim and Berkelbach^55^ presented the EOM-CC-like working equations for the *G*_0_*W*_0_ method in the Tamm–Dancoff approximation (*G*_0_*W*_0_-TDA) resulting in a frequency-independent method. More recently, the exact equivalence between the *G*_0_*W*_0_@HF method and the unitary IP/EA-EOM-CCSD method using the quasiboson approximation has been derived.^14^ We also note that different Green’s function methods have been formulated based on the CC formalism,^56−58^ offering an alternative avenue to compare CC and *GW* methods. The above relationships shall be further analyzed in future work to develop computationally efficient and accurate finite-size corrections for EOM-CCSD band gaps.

## 5 Conclusion

We have investigated the convergence of the EOM-CC band gap to the TDL by a formal analysis of the correlation structure factor of the IP and EA-EOM-CCSD method *S*^IP/EA^(**q**) in the long-wavelength limit (|**q**| → 0). As a result, we derived the convergence rate for the EOM-CC band gaps in the one, two, and three-dimensional case. These rates can be used in future extrapolations. However, the computed structure factors indicate that the asymptotic behavior can only be observed for relatively large super-cell sizes compared to the ground state correlation energies.

To verify the validity of the derived rates we compared to numerical results obtained for the one-dimensional case, using a chain of LiH unit cells and a chain of *trans*-polyacetylene as examples. The convergence to the TDL of the computed EOM-CC band gap for the chains is best fitted by *AN*_*k*_^–3^ ln(*N*_*k*_^–1^) for *N*_*k*_ → ∞. Our findings suggest that the two contributions for this limit [*N*_*k*_^–1^ ln(*N*_*k*_^–1^) and *N*_*k*_^–2^ ln(*N*_*k*_^–1^)], which in general are nonzero, can be neglected for these systems. This is directly related to the observation that the value and first derivative of the structure factor at the origin are very small in the studied systems. Assuming a generalization of this observation beyond the one-dimensional case can be made, we expect an *N*_*k*_^–3/2^ behavior for two-dimensional systems and an *N*_*k*_^–1^ behavior for three-dimensional ones. However, this requires that the same conditions for the structure factor are fulfilled for systems with higher dimensions.

Finally, we verified that our findings extend beyond EOM-CC theory and apply to the *G*_0_*W*_0_ method as well. We find that band gaps converge with the same rate in both theories, providing a formal justification to use the *GW* approach for the extrapolation of single-*k*-point EOM-CC results to the TDL.

## Supporting Information Available

Geometries of *trans*-polyacetylene and LiH used for the FHI-aims and VASP calculations (PDF)
