# Supplementary material for: Finite-Size Effects in Periodic EOM-CCSD for Ionization Energies and Electron Affinities: Convergence Rate and Extrapolation to the Thermodynamic Limit
Source: J Chem Theory Comput. 2025 Feb 4;21(4):1865–78. doi: 10.1021/acs.jctc.4c01451 (PMC11866753; doi:10.1021/acs.jctc.4c01451)
Supplement: Supplementary file 1 — ct4c01451_si_001.pdf [file ct4c01451_si_001.pdf]

Supporting Information: Finite-size Effects in periodic EOM-CCSD for Ionization Energies and Electron Affinities: Convergence Rate and Extrapolation to the Thermodynamic Limit

Evgeny Moerman,[*,†] Alejandro Gallo,[‡] Andreas Irmler,[‡] Tobias Schäfer,[‡] Felix Hummel,[‡] Andreas Grüneis,[‡] and Matthias Scheffler[†]

†*The NOMAD Laboratory at the FHI of the Max-Planck-Gesellschaft, Faradayweg 4–6, 14195 Berlin, Germany*

‡*Institute for Theoretical Physics, TU Wien, Wiedner Hauptstraße 8–10/136, 1040 Vienna, Austria*

E-mail: moerman@fhi-berlin.mpg.de

Structure of anisotropic lithium hydride supercells

Listing 1: POSCAR of LiH 1x1x10 supercell

```
LiH 1x1x10 supercell_exp
1.0
       2.8878240585        0.0000000000        0.0000000000
       1.4439120293        2.5009289963        0.0000000000
      14.4391202927        8.3364299878       23.5789847011
   Li    H
   10   10
```

```
Direct
     0.500000000         0.500000000         0.050000001
     0.500000000         0.500000000         0.150000006
     0.500000000         0.500000000         0.250000000
     0.500000000         0.500000000         0.349999994
     0.500000000         0.500000000         0.449999988
     0.500000000         0.500000000         0.550000012
     0.500000000         0.500000000         0.649999976
     0.500000000         0.500000000         0.750000000
     0.500000000         0.500000000         0.850000024
     0.500000000         0.500000000         0.949999988
     0.000000000         0.000000000         0.000000000
     0.000000000         0.000000000         0.100000001
     0.000000000         0.000000000         0.200000003
     0.000000000         0.000000000         0.300000012
     0.000000000         0.000000000         0.400000006
     0.000000000         0.000000000         0.500000000
     0.000000000         0.000000000         0.600000024
     0.000000000         0.000000000         0.699999988
     0.000000000         0.000000000         0.800000012
     0.000000000         0.000000000         0.899999976
```

Listing 2: POSCAR of LiH 1x1x50 supercell

```
LiH 1x1x50 supercell_exp
1.0
       2.8878240585       0.0000000000       0.0000000000
       1.4439120293       2.5009289963       0.0000000000
      72.1956024170      41.6821504898     117.8949250630
   Li    H
   50   50
Direct
     0.500000000         0.500000000         0.010000000
     0.500000000         0.500000000         0.029999999
     0.500000000         0.500000000         0.050000001
     0.500000000         0.500000000         0.070000000
     0.500000000         0.500000000         0.089999996
     0.500000000         0.500000000         0.109999999
     0.500000000         0.500000000         0.129999995
     0.500000000         0.500000000         0.150000006
     0.500000000         0.500000000         0.170000002
     0.500000000         0.500000000         0.189999998
     0.500000000         0.500000000         0.209999993
     0.500000000         0.500000000         0.230000004
     0.500000000         0.500000000         0.250000000
     0.500000000         0.500000000         0.270000011
     0.500000000         0.500000000         0.289999992
     0.500000000         0.500000000         0.310000002
     0.500000000         0.500000000         0.330000013
```

0.500000000	0.500000000	0.349999994
0.500000000	0.500000000	0.370000005
0.500000000	0.500000000	0.389999986
0.500000000	0.500000000	0.409999996
0.500000000	0.500000000	0.430000007
0.500000000	0.500000000	0.449999988
0.500000000	0.500000000	0.469999999
0.500000000	0.500000000	0.490000010
0.500000000	0.500000000	0.509999990
0.500000000	0.500000000	0.529999971
0.500000000	0.500000000	0.550000012
0.500000000	0.500000000	0.569999993
0.500000000	0.500000000	0.589999974
0.500000000	0.500000000	0.610000014
0.500000000	0.500000000	0.629999995
0.500000000	0.500000000	0.649999976
0.500000000	0.500000000	0.670000017
0.500000000	0.500000000	0.689999998
0.500000000	0.500000000	0.709999979
0.500000000	0.500000000	0.730000019
0.500000000	0.500000000	0.750000000
0.500000000	0.500000000	0.769999981
0.500000000	0.500000000	0.790000021
0.500000000	0.500000000	0.810000002
0.500000000	0.500000000	0.829999983
0.500000000	0.500000000	0.850000024
0.500000000	0.500000000	0.870000005
0.500000000	0.500000000	0.889999986
0.500000000	0.500000000	0.910000026
0.500000000	0.500000000	0.930000007
0.500000000	0.500000000	0.949999988
0.500000000	0.500000000	0.970000029
0.500000000	0.500000000	0.990000010
0.000000000	0.000000000	0.000000000
0.000000000	0.000000000	0.020000000
0.000000000	0.000000000	0.039999999
0.000000000	0.000000000	0.059999999
0.000000000	0.000000000	0.079999998
0.000000000	0.000000000	0.100000001
0.000000000	0.000000000	0.119999997
0.000000000	0.000000000	0.140000001
0.000000000	0.000000000	0.159999996
0.000000000	0.000000000	0.180000007
0.000000000	0.000000000	0.200000003
0.000000000	0.000000000	0.219999999
0.000000000	0.000000000	0.239999995
0.000000000	0.000000000	0.259999990
0.000000000	0.000000000	0.280000001
0.000000000	0.000000000	0.300000012
0.000000000	0.000000000	0.319999993

```
    0.000000000         0.000000000         0.340000004
    0.000000000         0.000000000         0.360000014
    0.000000000         0.000000000         0.379999995
    0.000000000         0.000000000         0.400000006
    0.000000000         0.000000000         0.419999987
    0.000000000         0.000000000         0.439999998
    0.000000000         0.000000000         0.460000008
    0.000000000         0.000000000         0.479999989
    0.000000000         0.000000000         0.500000000
    0.000000000         0.000000000         0.519999981
    0.000000000         0.000000000         0.540000021
    0.000000000         0.000000000         0.560000002
    0.000000000         0.000000000         0.579999983
    0.000000000         0.000000000         0.600000024
    0.000000000         0.000000000         0.620000005
    0.000000000         0.000000000         0.639999986
    0.000000000         0.000000000         0.660000026
    0.000000000         0.000000000         0.680000007
    0.000000000         0.000000000         0.699999988
    0.000000000         0.000000000         0.720000029
    0.000000000         0.000000000         0.740000010
    0.000000000         0.000000000         0.759999990
    0.000000000         0.000000000         0.779999971
    0.000000000         0.000000000         0.800000012
    0.000000000         0.000000000         0.819999993
    0.000000000         0.000000000         0.839999974
    0.000000000         0.000000000         0.860000014
    0.000000000         0.000000000         0.879999995
    0.000000000         0.000000000         0.899999976
    0.000000000         0.000000000         0.920000017
    0.000000000         0.000000000         0.939999998
    0.000000000         0.000000000         0.959999979
    0.000000000         0.000000000         0.980000019
```

Listing 3: POSCAR of LiH 1x1x100 supercell

```
LiH 1x1x100 supercell_exp
1.0
        2.8878240585        0.0000000000        0.0000000000
        1.4439120293        2.5009289963        0.0000000000
      144.3912048340       83.3643009795      235.7898501260
   Li    H
  100  100
Direct
    0.500000000         0.500000000         0.005000000
    0.500000000         0.500000000         0.015000000
    0.500000000         0.500000000         0.025000000
    0.500000000         0.500000000         0.035000000
    0.500000000         0.500000000         0.044999998
```

0.500000000	0.500000000	0.055000000
0.500000000	0.500000000	0.064999998
0.500000000	0.500000000	0.075000003
0.500000000	0.500000000	0.085000001
0.500000000	0.500000000	0.094999999
0.500000000	0.500000000	0.104999997
0.500000000	0.500000000	0.115000002
0.500000000	0.500000000	0.125000000
0.500000000	0.500000000	0.135000005
0.500000000	0.500000000	0.144999996
0.500000000	0.500000000	0.155000001
0.500000000	0.500000000	0.165000007
0.500000000	0.500000000	0.174999997
0.500000000	0.500000000	0.185000002
0.500000000	0.500000000	0.194999993
0.500000000	0.500000000	0.204999998
0.500000000	0.500000000	0.215000004
0.500000000	0.500000000	0.224999994
0.500000000	0.500000000	0.234999999
0.500000000	0.500000000	0.245000005
0.500000000	0.500000000	0.254999995
0.500000000	0.500000000	0.264999986
0.500000000	0.500000000	0.275000006
0.500000000	0.500000000	0.284999996
0.500000000	0.500000000	0.294999987
0.500000000	0.500000000	0.305000007
0.500000000	0.500000000	0.314999998
0.500000000	0.500000000	0.324999988
0.500000000	0.500000000	0.335000008
0.500000000	0.500000000	0.344999999
0.500000000	0.500000000	0.354999989
0.500000000	0.500000000	0.365000010
0.500000000	0.500000000	0.375000000
0.500000000	0.500000000	0.384999990
0.500000000	0.500000000	0.395000011
0.500000000	0.500000000	0.405000001
0.500000000	0.500000000	0.414999992
0.500000000	0.500000000	0.425000012
0.500000000	0.500000000	0.435000002
0.500000000	0.500000000	0.444999993
0.500000000	0.500000000	0.455000013
0.500000000	0.500000000	0.465000004
0.500000000	0.500000000	0.474999994
0.500000000	0.500000000	0.485000014
0.500000000	0.500000000	0.495000005
0.500000000	0.500000000	0.504999995
0.500000000	0.500000000	0.514999986
0.500000000	0.500000000	0.524999976
0.500000000	0.500000000	0.535000026
0.500000000	0.500000000	0.545000017

0.500000000	0.500000000	0.555000007
0.500000000	0.500000000	0.564999998
0.500000000	0.500000000	0.574999988
0.500000000	0.500000000	0.584999979
0.500000000	0.500000000	0.595000029
0.500000000	0.500000000	0.605000019
0.500000000	0.500000000	0.615000010
0.500000000	0.500000000	0.625000000
0.500000000	0.500000000	0.634999990
0.500000000	0.500000000	0.644999981
0.500000000	0.500000000	0.654999971
0.500000000	0.500000000	0.665000021
0.500000000	0.500000000	0.675000012
0.500000000	0.500000000	0.685000002
0.500000000	0.500000000	0.694999993
0.500000000	0.500000000	0.704999983
0.500000000	0.500000000	0.714999974
0.500000000	0.500000000	0.725000024
0.500000000	0.500000000	0.735000014
0.500000000	0.500000000	0.745000005
0.500000000	0.500000000	0.754999995
0.500000000	0.500000000	0.764999986
0.500000000	0.500000000	0.774999976
0.500000000	0.500000000	0.785000026
0.500000000	0.500000000	0.795000017
0.500000000	0.500000000	0.805000007
0.500000000	0.500000000	0.814999998
0.500000000	0.500000000	0.824999988
0.500000000	0.500000000	0.834999979
0.500000000	0.500000000	0.845000029
0.500000000	0.500000000	0.855000019
0.500000000	0.500000000	0.865000010
0.500000000	0.500000000	0.875000000
0.500000000	0.500000000	0.884999990
0.500000000	0.500000000	0.894999981
0.500000000	0.500000000	0.904999971
0.500000000	0.500000000	0.915000021
0.500000000	0.500000000	0.925000012
0.500000000	0.500000000	0.935000002
0.500000000	0.500000000	0.944999993
0.500000000	0.500000000	0.954999983
0.500000000	0.500000000	0.964999974
0.500000000	0.500000000	0.975000024
0.500000000	0.500000000	0.985000014
0.500000000	0.500000000	0.995000005
0.000000000	0.000000000	0.000000000
0.000000000	0.000000000	0.010000000
0.000000000	0.000000000	0.020000000
0.000000000	0.000000000	0.029999999
0.000000000	0.000000000	0.039999999

0.000000000	0.000000000	0.050000001
0.000000000	0.000000000	0.059999999
0.000000000	0.000000000	0.070000000
0.000000000	0.000000000	0.079999998
0.000000000	0.000000000	0.090000004
0.000000000	0.000000000	0.100000001
0.000000000	0.000000000	0.109999999
0.000000000	0.000000000	0.119999997
0.000000000	0.000000000	0.129999995
0.000000000	0.000000000	0.140000001
0.000000000	0.000000000	0.150000006
0.000000000	0.000000000	0.159999996
0.000000000	0.000000000	0.170000002
0.000000000	0.000000000	0.180000007
0.000000000	0.000000000	0.189999998
0.000000000	0.000000000	0.200000003
0.000000000	0.000000000	0.209999993
0.000000000	0.000000000	0.219999999
0.000000000	0.000000000	0.230000004
0.000000000	0.000000000	0.239999995
0.000000000	0.000000000	0.250000000
0.000000000	0.000000000	0.259999990
0.000000000	0.000000000	0.270000011
0.000000000	0.000000000	0.280000001
0.000000000	0.000000000	0.289999992
0.000000000	0.000000000	0.300000012
0.000000000	0.000000000	0.310000002
0.000000000	0.000000000	0.319999993
0.000000000	0.000000000	0.330000013
0.000000000	0.000000000	0.340000004
0.000000000	0.000000000	0.349999994
0.000000000	0.000000000	0.360000014
0.000000000	0.000000000	0.370000005
0.000000000	0.000000000	0.379999995
0.000000000	0.000000000	0.389999986
0.000000000	0.000000000	0.400000006
0.000000000	0.000000000	0.409999996
0.000000000	0.000000000	0.419999987
0.000000000	0.000000000	0.430000007
0.000000000	0.000000000	0.439999998
0.000000000	0.000000000	0.449999988
0.000000000	0.000000000	0.460000008
0.000000000	0.000000000	0.469999999
0.000000000	0.000000000	0.479999989
0.000000000	0.000000000	0.490000010
0.000000000	0.000000000	0.500000000
0.000000000	0.000000000	0.509999990
0.000000000	0.000000000	0.519999981
0.000000000	0.000000000	0.529999971
0.000000000	0.000000000	0.540000021

0.000000000	0.000000000	0.550000012
0.000000000	0.000000000	0.560000002
0.000000000	0.000000000	0.569999993
0.000000000	0.000000000	0.579999983
0.000000000	0.000000000	0.589999974
0.000000000	0.000000000	0.600000024
0.000000000	0.000000000	0.610000014
0.000000000	0.000000000	0.620000005
0.000000000	0.000000000	0.629999995
0.000000000	0.000000000	0.639999986
0.000000000	0.000000000	0.649999976
0.000000000	0.000000000	0.660000026
0.000000000	0.000000000	0.670000017
0.000000000	0.000000000	0.680000007
0.000000000	0.000000000	0.689999998
0.000000000	0.000000000	0.699999988
0.000000000	0.000000000	0.709999979
0.000000000	0.000000000	0.720000029
0.000000000	0.000000000	0.730000019
0.000000000	0.000000000	0.740000010
0.000000000	0.000000000	0.750000000
0.000000000	0.000000000	0.759999990
0.000000000	0.000000000	0.769999981
0.000000000	0.000000000	0.779999971
0.000000000	0.000000000	0.790000021
0.000000000	0.000000000	0.800000012
0.000000000	0.000000000	0.810000002
0.000000000	0.000000000	0.819999993
0.000000000	0.000000000	0.829999983
0.000000000	0.000000000	0.839999974
0.000000000	0.000000000	0.850000024
0.000000000	0.000000000	0.860000014
0.000000000	0.000000000	0.870000005
0.000000000	0.000000000	0.879999995
0.000000000	0.000000000	0.889999986
0.000000000	0.000000000	0.899999976
0.000000000	0.000000000	0.910000026
0.000000000	0.000000000	0.920000017
0.000000000	0.000000000	0.930000007
0.000000000	0.000000000	0.939999998
0.000000000	0.000000000	0.949999988
0.000000000	0.000000000	0.959999979
0.000000000	0.000000000	0.970000029
0.000000000	0.000000000	0.980000019
0.000000000	0.000000000	0.990000010

Structure of *trans*-Polyacetylene (tPA)

Listing 4: geometry.in of tPA unit cell used for FHI-aims calculations

```
lattice_vector 2.461964 0.000000 0.000000
lattice_vector 0.000000 80.033830 0.000000
lattice_vector -0.474919 0.000000 99.817314

atom_frac 0.018380 0.500000 0.503244 C
atom_frac 0.020653 0.500000 0.514117 H
atom_frac 0.501848 0.500000 0.496756 C
atom_frac 0.499598 0.500000 0.485882 H
```

Listing 5: POSCAR of tPA unit cell used for VASP calculations

```
tPA_1x1x1_vasp
1.0
   2.463009          0.000000          0.000000
   0.000000          8.003383          0.000000
   0.000000          0.000000          9.986052
   C    H
   2   2
Direct
     0.997380          0.000000          0.967580
     0.482582          0.000000          0.032413
     0.997345          0.000000          0.858860
     0.482214          0.000000          0.141123
```

Listing 6: geometry.in of the tPA3 unit cell

```
lattice_vector    2.44924228     0.00000000     0.00000000
lattice_vector    0.00000000     90.0000000     0.00000000
lattice_vector    0.00000000    -0.00000000    90.0000000
atom 1.8546827242997082 5.339047178117827 0.0 C
atom 3.033575443779031 4.660952821882173 0.0 C
atom 1.862431931370947 6.429019631801757 0.0 H
atom 3.025826236707793 3.570980368198243 0.0 H
```
